# Practitioners' Bookshelf

**Published:** 1892-07-16

**Authors:** 


### PRACTITIONERS' BOOKSHELF.

### OUTLINES OF INSANITY.*

This book is designed for the use of medical practitioners, Justices of the Peace, and asylum managers, and is an attempt to present in a concise form the salient features of mental disorder. In the opening chapter the processes of normal mentation are discussed very briefly and in simple language, and suitably leads up to the following chapters, which treat of the chief varieties of mental aberration. Of course, it is an extremely difficult matter to convey to beginners in psychology anything like a clear idea of the nature of mental processes in fifteen pages, and we think that Dr. Walmsley would do well in future editions to extend this chapter, since, without a knowledge of mental physiology, it is clearly impossible to understand the true nature of

* "Outlines of Insanity." By F. H. Walmsley, M.D. 8vo., pp. 154. (London: The Scientific Press (Limited). 1892.)

mental pathology. This chapter is too metaphysical, and would be more intelligible if it approached the subject from its physiological aspects. The chapters on abnormal mentation and the various forms of insanity give a sufficiently clear idea of the various types discussed, and cannot fail to be of great service to Justices of the Peace who have themselves to visit alleged lunatics, and for medical men who have not had that practical training in insanity which in future will be demanded of all those who desire to become qualified for practice. A perusal of the Appendix III., on actions in lunacy against medical men, shows the very unfair position in which they may be placed if they have the temerity to do their duty to the public in certifying to the insanity, even after the most careful and painstaking investigation, of the cases brought under their notice. It supplies information that heretofore magistrates and many practitioners must have greatly desired.

### THE HARVARD MEDICAL SCHOOL.

This pamphlet,* which has been sent us for review, bears an interesting record of the foundation of the Harvard Medical School Association, an institution which, in the words of Dr. Bowditch, has for its object the banding together of those who have drawn their intellectual nourishment from a common source for the purposes of mutual encouragement and good fellowship.

In England we have so few opportunities of learning anything about the medical schools on the other side of the Atlantic that it is very refreshing to read from the pages of this pamphlet that there is as much enthusiasm and *esprit de corps* among the alumni of this medical school as is the case with the older institutions in the mother country.

The actual number of alumni enrolled in the records of the association is 750, a total which the promoters may well be proud of, but prouder still must they feel at numbering in the grand total such names as Bowditch, Bigelow, and Holmes, names, indeed, not only familiar amongst the English-speaking races, but wherever surgery and medicine are taught, though few perhaps are aware that all of these names lend lustre to the medical school of Harvard University.

At the first annual dinner, held in commemoration of the foundation of the association, some hundred and fifty newly-enrolled alumni sat down together and congratulated themselves on the foundation of so promising an institution. Amongst the letters of regret which were read at the dinner from those of the alumni who were unable to be present was one from Dr. Oliver Wendell Holmes; so characteristic in style is it that we cannot refrain from quoting a few lines. After expressing his regrets at not being able to be present, he continues:—

"I know the members would receive me kindly as a relic of the past, not without a certain value as a fragment of antiquity. I have long been the sole survivor of that primeval faculty of which I became a member in 1847. In that point of view I am not merely a rarity, I am a unique specimen, and have an adventitious market price, like one of those rare cents for which collectors pay a premium; half worn-out old copper as it is, its scarcity makes it worth a dime or, perhaps, a dollar."

Institutions of this sort in American universities, whose value in the mother country has long been felt, have our best wishes for a successful and prosperous career, that of Harvard University, commencing as it does under such favourable auspices, cannot be otherwise than a success.

The extension of study for the degree of Bachelor of Medicine at Harvard University from three years to four has our heartiest sympathy. This extension is contemporaneous with the founding of this new society, and if in a few years, the course be further extended to five years, it will be of undoubted advantage both to the medical school and the American public.

* "Bulletin of the Harvard Medical School Association. No. 1. 1891."

